# Diagnostic accuracy of artificial intelligence models for imaging detection of hepatic steatosis through systematic review and meta analysis

**DOI:** 10.1038/s41598-025-17386-3

**Published:** 2025-10-02

**Authors:** V. Nivethitha, Roy Arokiam Daniel, Aninda Debnath, Vignesh Dwarakanathan, Girish Jeer, G. Kavipriya

**Affiliations:** 1 School of Computer Science Engineering (SCOPE), Vellore Institute of Technology (VIT), Chennai, 600 127 Tamil Nadu India; 2 Department of Community Medicine, ESIC Medical College and Hospital, KK Nagar, Chennai, 600 078 Tamil Nadu India; 3 Department of Community Medicine, Maulana Azad Medical College, Delhi, India; 4 Centre for Community Medicine, All India Institute of Medical Sciences (AIIMS), New Delhi, India

**Keywords:** Non-alcoholic fatty liver disease, Artificial intelligence, Convolutional neural network, Diagnostic accuracy, Systematic review, Meta-analysis, Computational biology and bioinformatics, Gastroenterology, Medical research

## Abstract

Non-alcoholic fatty liver disease (NAFLD) is an increasingly prevalent chronic liver condition affecting nearly 30% of the global population. Characterized by hepatic steatosis in the absence of significant alcohol intake, NAFLD can progress to non-alcoholic steatohepatitis (NASH), fibrosis, cirrhosis, and hepatocellular carcinoma. Although liver biopsy is the diagnostic gold standard, its invasiveness, cost, and associated risks limit widespread application. Artificial intelligence (AI) offers promising non-invasive alternatives by leveraging large datasets to enhance diagnostic precision. To evaluate the diagnostic accuracy of artificialintelligence algorithms for the imaging detection of hepatic steatosis, the essential first step in the metabolic dysfunctionassociated steatotic liver disease (MASLD) spectrum. A comprehensive literature search was conducted across PubMed, Scopus, Embase, Cochrane Library, and Google Scholar for studies published between January 2016 and January 2025. Studies were included if they involved adult populations, employed AI algorithms for NAFLD diagnosis, and reported sufficient diagnostic accuracy measures. Quality assessment was performed using the QUADAS-2 tool. Meta-analysis was conducted using a bivariate random-effects model to estimate pooled sensitivity, specificity, and area under the hierarchical summary receiver operating characteristic (HSROC) curve. Out of 29 studies included in the systematic review, 19 met the criteria for meta-analysis, comprising a total of 344,266 participants. AI-based diagnostic models showed excellent performance, with pooled sensitivity of 91% (95% CI: 84–95%), specificity of 92% (95% CI: 86–96%), and an AUC of 0.97 (95% CI: 0.95–0.98). The diagnostic odds ratio was 123.7, indicating high discriminatory capacity. Convolutional neural networks (CNNs) demonstrated superior accuracy (AUC = 1.00) compared to other AI classifiers. Subgroup analysis revealed higher diagnostic accuracy in studies validated with imaging standards compared to those using liver biopsy. Model performance was also influenced by the type of classifier and validation method used. AI-based models, particularly CNNs, exhibit high diagnostic accuracy for detecting hepatic steatosis and offer promising non-invasive alternatives to traditional modalities. These tools have the potential to transform early detection and screening, especially in resource-limited settings. Future research should focus on external validation, multicentric trials, and standardized reporting for clinical integration.

**Supplementary Information:**

The online version contains supplementary material available at 10.1038/s41598-025-17386-3.

## Introduction

Nonalcoholic fatty liver disease (NAFLD) has emerged as a significant global health issue that currently affects approximately 32% of the world’s population. NAFLD represents a spectrum of liver conditions, ranging from simple hepatic steatosis to more severe nonalcoholic steatohepatitis (NASH). NASH can progress to liver cirrhosis, liver failure, or hepatocellular carcinoma if left untreated. The increasing prevalence of metabolic syndrome has been strongly linked to the increase in NAFLD cases^1^. Given the potential for severe health consequences, early diagnosis is critical for preventing disease progression and managing patient outcomes effectively.

The current diagnostic landscape for NAFLD faces several challenges. Although liver biopsy is considered the gold standard for definitive assessment of liver fat content and fibrosis staging, it is invasive, costly, and carries risks such as bleeding and infection, limiting its suitability for large-scale screening or routine monitoring. Noninvasive alternatives such as ultrasound and magnetic resonance imaging (MRI) are more commonly used but have notable limitations^2^. Ultrasound, while widely available and cost-effective, is highly operator-dependent and lacks sensitivity, particularly in detecting early-stage NAFLD or obese individuals, with sensitivity ranging from 53 to 76% and specificity ranging from 76 to 93%^3,4^. MRI, especially with advanced techniques such as proton density fat fraction (PDFF) and magnetic resonance elastography (MRE), offers higher diagnostic accuracy, with sensitivity ranging from 76 to 90% and specificity ranging from 87 to 91%^5^. However, its high cost and limited accessibility make it impractical for widespread use, particularly in resource-constrained settings where NAFLD prevalence is increasing. These limitations highlight the urgent need for innovative, scalable, and noninvasive diagnostic tools to improve NAFLD detection through estimation of hepatic steatosis and management, especially in low-resource healthcare environments.

In 2023, international hepatology societies updated the nomenclature from nonalcoholic fatty liver disease (NAFLD) to metabolic dysfunction‑associated steatotic liver disease (MASLD), defined by the presence of hepatic steatosis plus at least one metabolic risk factor such as obesity, dyslipidemia, or type 2 diabetes^6^. All studies included in our review were conducted under the former NAFLD criteria. Nevertheless, the imaging hallmarks of steatosis remain unchanged, and our pooled estimates of diagnostic accuracy thus apply equally to MASLD. Because MASLD requires hepatic steatosis together with at least one metabolic risk factor and the exclusion of competing aetiologies, imaging alone can identify the steatosis component but cannot furnish a definitive MASLD diagnosis; our review therefore focuses specifically on AIbased detection of hepatic fat.

Artificial intelligence (AI) offers a promising solution to these diagnostic challenges. Machine learning (ML) and deep learning (DL) algorithms, especially convolutional neural networks (CNNs), can identify complex, high-dimensional imaging patterns that are often imperceptible to human observers. CNNs have shown excellent performance in detecting hepatic steatosis and staging NAFLD via ultrasound imaging, whereas recurrent neural networks (RNNs) have been effective in predicting disease trajectories via longitudinal clinical data^7^. By enhancing both sensitivity and specificity, AI has the potential to bridge existing diagnostic gaps, particularly in early-stage disease, thereby improving clinical decision-making. Its utility is especially relevant in resource-limited environments, where access to advanced imaging, such as MRI, is restricted, but ultrasound is widely available^8^.

The global relevance of AI in NAFLD diagnostics through the estimation of heaptic steatosis is particularly significant for low- and middle-income countries, where the prevalence of obesity and metabolic syndrome is rising rapidly. In rural and underserved regions with limited access to advanced diagnostic tools such as MRI, AI integration into mobile health platforms and telemedicine can democratize NAFLD diagnostics. By enhancing the diagnostic capabilities of commonly available tools such as ultrasound, AI has the potential to enable early detection and reduce the long-term healthcare burden of NAFLD in such settings. This systematic review and meta-analysis aim to estimate the diagnostic accuracy of AI models in detecting hepatic steatosis compared with established methods (USG, MRI and liver biopsy). Additionally, the study examines the impact of different AI models and diagnostic standards on performance. These findings provide critical insights into the role of AI in NAFLD through the estimation of hepatic steatosis and inform future research in this field.

## Materials and methods

### Literature search strategy

A comprehensive literature search was conducted across PubMed, Scopus, Embase, Google Scholar and the Cochrane Library. The search strategy included a combination of Medical Subject Headings (MeSH) and free-text terms: “Nonalcoholic Fatty Liver Disease” OR “NAFLD” OR “MASLD” OR “hepatic steatosis” AND “Artificial Intelligence” OR “Machine Learning” OR “Deep Learning” AND “Diagnosis” AND (“Ultrasound” OR “MRI” OR “Liver Biopsy”). Boolean operators (AND, OR) were used to optimize the sensitivity (Table S1). Manual reference screening was also performed. The search was restricted to articles published between January 2016 and January 2025 to ensure the inclusion of the most recent and methodologically robust applications of artificial intelligence in NAFLD diagnosis. This 10-year window was chosen to capture the evolution and clinical integration of modern AI techniques, particularly deep learning models, which have gained substantial traction only in the past decade. No language restrictions were applied. The protocol was registered with PROSPERO (CRD42024582236), and the review followed the PRISMA 2020 guidelines^9^ and the Cochrane Handbook.

### Selection criteria

The inclusion criteria for this study were as follows: (1) studies conducted among adult populations (aged 18 years and above) with suspected or confirmed nonalcoholic fatty liver disease (NAFLD), nonalcoholic steatohepatitis (NASH), or metabolic-associated fatty liver disease (MAFLD); (2) studies utilizing artificial intelligence (AI) algorithms, including machine learning models such as neural networks, support vector machines, random forests, and deep learning, applied to imaging data (ultrasound, MRI, CT) or clinical parameters (e.g., blood tests, clinical data) for the diagnosis of NAFLD; (3) studies reporting diagnostic accuracy metrics, including sensitivity, specificity, positive predictive value (PPV), negative predictive value (NPV), and area under the curve (AUC); and (4) original research articles published between 2016 and 2025. The exclusion criteria were as follows: (1) studies focused solely on animal models or in vitro analyses; (2) conference abstracts lacking detailed methodologies or results; (3) reviews or editorials; and (4) studies that did not provide sufficient data to construct 2 × 2 contingency tables.

#### Study selection and data extraction

Two independent reviewers (RAD and AD) initially screened the titles of all records retrieved from the database search. Abstracts of potentially relevant studies were then assessed, and those meeting the predefined inclusion criteria were selected. Disagreements at any stage were resolved through discussion, with arbitration by a third reviewer (NV) when needed. Duplicate records were identified and removed prior to full-text review. Full-text articles corresponding to the selected abstracts were retrieved and assessed for final inclusion based on eligibility criteria. In addition, reference lists of all included studies were manually screened to identify any further relevant articles that may have been missed in the primary search.

A standardized data extraction form was developed and pilot-tested on a subset of five studies to ensure clarity and consistency. After refinement, two independent reviewers used the finalized form to extract data. Information collected included study characteristics (first author, year of publication, country), participant demographics (mean age, sex distribution, NAFLD prevalence), details of AI models used (e.g., convolutional neural networks [CNNs], recurrent neural networks [RNNs]), imaging modalities employed/reference standard, and diagnostic performance metrics such as sensitivity, specificity, positive predictive value (PPV), negative predictive value (NPV), true positives (TP), false positives (FP), true negatives (TN), and false negatives (FN). Inter-reviewer reliability for study selection and data extraction was quantified using Cohen’s kappa statistic, with values > 0.80 interpreted as excellent agreement. Any disagreements were resolved by consensus or consultation with the third reviewer. For the assessment of hepatic steatosis, histology and magnetic resonance proton density fat fraction (MRI‑PDFF) were considered the highest-quality reference standards. Studies that used ultrasound or computed tomography as both the index and reference test were retained but categorized as employing an “imaging-only reference.” These were deemed to carry a higher risk of bias in the QUADAS reference standard domain.

### Risk of bias assessment

The methodological quality of the included studies was assessed via the Quality Assessment of Diagnostic Accuracy Studies-2 (QUADAS-2) tool^10^, which is specifically designed for evaluating diagnostic accuracy in systematic reviews. This tool assesses the risk of bias across four domains: patient selection, index test, reference standard, and flow and timing. Each domain was rated as having low risk, high risk, or some concerns of bias. Two independent reviewers (RAD and AD) conducted the risk of bias assessment for each study. To minimize bias, the reviewers were blinded to each other’s ratings during the initial assessment, and any discrepancies were resolved through discussion with a third reviewer (NV). The overall assessments were visualized via the robvis R package^11^, which generated both traffic light plots and summary bar charts to enhance transparency. Studies with a high risk of bias in one or more domains were not excluded from the meta-analysis; however, they were incorporated into sensitivity analyses to assess their influence on the pooled estimates. This ensured that the final conclusions were robust to potential methodological variability and that no informative studies were omitted solely on the basis of bias concerns.

### Statistical analysis

The meta-analysis was performed via Stata version 18 (StataCorp, College Station, TX, USA)^12^. Pooled estimates of sensitivity, specificity, and diagnostic odds ratios (DORs) were calculated via a bivariate random effects model, which accounts for both within- and between-study variability and is particularly suitable for diagnostic accuracy studies. The *midas*, *metandi*, and *midasplot* commands were used to perform these analyses and generate corresponding forest plots and hierarchical summary receiver operating characteristic (HSROC) curves. The area under the HSROC curve (AUC), with 95% confidence intervals, was used as a global measure of diagnostic performance. For studies with zero cell counts (e.g., 0 true positives or 0 false positives), a continuity correction of 0.5 was applied to allow for stable variance estimation and to include such studies in the pooled analysis without distortion. Heterogeneity across studies was assessed via the I² statistic, with values greater than 50% interpreted as indicating substantial heterogeneity. To explore sources of heterogeneity, prespecified subgroup analyses were conducted on the basis of (i) the AI classifier, (ii) the reference standard, (iii) NAFLD status, and (iv) the validation method. Sensitivity analysis will be performed on the basis of the influence analysis after removing the outliers. To examine the effect of study size, we repeated the bivariate model after excluding studies whose training cohort comprised fewer than 500 participants. Publication bias was assessed both qualitatively via visual inspection of Deeks’ funnel plots and quantitatively via Deeks’ asymmetry test, with a p value < 0.10 indicating potential small-study effects.

### Ethical considerations

As this study involved a meta-analysis of previously published data, no primary data collection was undertaken. Therefore, no ethical approval or patient consent was needed. All included studies were assumed to have adhered to ethical guidelines such as obtaining Institutional Review Board (IRB) approval and informed consent from participants, in line with the Declaration of Helsinki.

## Results

### Study selection

**Fig. 1:**
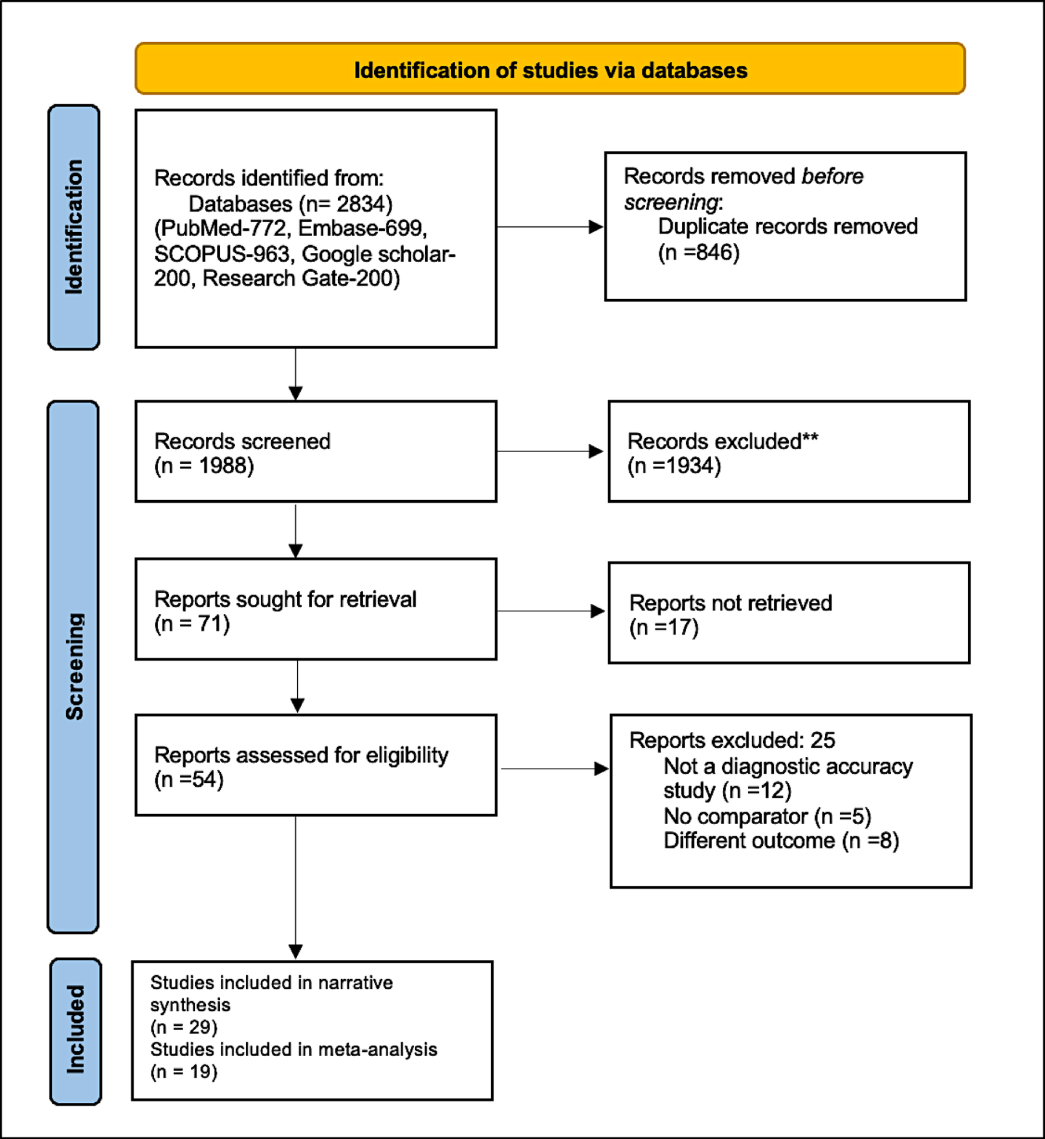
Flow chart of studies included in the systematic review and meta-analysis.

A total of 2,834 records were identified through database searches, including PubMed (772), Embase (699), SCOPUS (963), Google Scholar (200), and ResearchGate (200). After the removal of 846 duplicate records, 1,988 records were screened on the basis of titles and abstracts, leading to the exclusion of 1,934 records that did not meet the inclusion criteria. A total of 71 reports were subsequently sought for full-text retrieval; however, 17 reports could not be accessed. Among the 54 full-text articles assessed for eligibility, 25 were excluded for the following reasons: 12 were not diagnostic accuracy studies, 5 lacked a comparator, and 8 reported different outcomes. Finally, 29 studies were included in the narrative synthesis, 19 of which were eligible for inclusion in the meta-analysis, as shown in Fig. 1. This systematic selection process adhered to PRISMA guidelines to ensure the inclusion of high-quality and relevant studies. The inter-reviewer agreement for title and abstract screening was strong, with a Cohen’s kappa value of 0.84, indicating excellent consistency. Disagreements occurred in 9% of the cases and were resolved through consensus. The final inclusion rate following title and abstract screening was 1.46%, indicating rigorous eligibility criteria and high screening specificity.

### Characteristics of the studies included in the systematic review

A total of 29 studies published between 2016 and 2024 were included in this systematic review. The geographic distribution of studies was diverse, with the largest proportion originating from China (31%), followed by the United States (17%), reflecting a growing global interest in artificial intelligence (AI) applications for NAFLD detection. The included studies investigated adult populations with either suspected or confirmed nonalcoholic fatty liver disease (NAFLD), incorporating both retrospective and prospective study designs. Collectively, these studies included a pooled sample size of 344,266 individuals, with a mean participant age of 47.3 years and a female representation of approximately 51.6%. Logistic regression emerged as the most frequently used AI classifier (44.8%), followed by convolutional neural networks (CNNs), random forests (RFs), and support vector machines (SVMs). Several studies have employed hybrid or ensemble approaches, reflecting the increasing complexity and adaptability of AI models in clinical diagnostics.

In terms of validation techniques, 10-fold cross-validation was the most commonly employed strategy (25%), whereas external validation using independent datasets was reported in 8 studies (27.6%). However, 21% of the studies did not specify their validation methodology, underscoring the need for improved reporting standards in AI-based diagnostic research. The reference standards used to define NAFLD status included ultrasound (USG) in 51.7% of the studies, followed by liver biopsy and magnetic resonance imaging (MRI). Among studies using biopsy, only a few explicitly mentioned pathologist-blinded verification; similarly, in imaging-based studies, radiologist blinding was rarely reported. This highlights a potential risk of verification bias in reference standard ascertainment, which was accounted for in the risk of bias assessment. Diagnostic performance across the studies was generally high. The sensitivity values ranged from 55 to 99.7%, whereas the specificity values ranged from 64.9 to 94%. The area under the curve (AUC) values ranged from 73 to 99.9%, with most studies reporting values above 85%, indicating excellent model discrimination. These performance metrics suggest that AI tools hold substantial promise for assisting NAFLD detection, particularly in contexts where access to liver biopsy or MRI is limited. A comprehensive summary of these characteristics is presented in Table 1 (Main Manuscript), and a more detailed breakdown is provided in Table S2.

**Table 1:** Summary of the studies included in the systematic review.

Author	Publication year	Country	Sample Size	Mean/Median Age	Proportion of Females (%)	Classifier	Reference standard	TP	TN	FP	FN
Corey et al.^13^	2016	USA	620	61.6	0.56	Logistic regression	Liver Biopsy	161	276	27	155
Yip et al.^14^	2017	Hong Kong	922	48.1	0.57	Logistic regression	MRI	243	592	66	21
Kuppili et al.^15^	2017	Portugal	63	43	0.5	Logistic regression	USG	33	25	2	3
Biswas et al.^16^	2018	Portugal	101	50	0.48	Logistic regression	USG	36	27	0	0
Byra et al.^17^	2018	Poland	55	40.1	0.2	CNN	Liver Biopsy	38	15	3	0
Islam et al.^18^	2018	Taiwan	994	62	0.536	Random forest	USG	439	260	141	154
Ma et al.^19^	2018	China	10,508	50	0.24	Logistic regression	USG	1702	7012	974	820
Wu et al.^20^	2018	Taiwan	577	54	0.549	Random forest	USG	329	172	28	48
Canbay et al.^21^	2019	Germany	286	43.5	0.76	Logistic regression	Liver Biopsy	-			
Cao et al.^22^	2019	China	240	41	0.45	Logistic regression	USG	-			
Munsterman et al.^23^	2019	Netherlands	79			Random forest	MRI	-			
Shi et al.^24^	2019	China	60	41	0.36	Logistic regression	USG	30	24	2	4
Han et al.^25^	2020	USA	204	52	0.6	CNN	MRI	97	30	2	2
Pasdar et al.^26^	2020	UK	1514	57		Random forest	MRI	677	2668	838	334
Agarwal et al.^27^	2021	USA	1016	48.3	0.615	Random forest	Liver Biopsy	-			
Qu et al.^28^	2021	USA	87			Logistic regression	Clinical data	-			
Liu et al.^29^	2021	China	3310			Random forest	Liver Biopsy	1136	2802	280	724
Lim et al.^30^	2021	South Korea	1652	31.4	0.337	Logistic regression	Liver Biopsy	39	1298	304	11
Rhyou et al.^31^	2021	South Korea	3200			Logistic regression	USG	1063	2133	0	3
Constantinescu et al.^32^	2021	Romania	629		0.52	CNN	USG	62	61	2	8
Zamanian et al.^33^	2021	Poland	55	40	0.8	CNN	Liver Biopsy	70	74	0	2
Chen et al.^34^	2021	China	7396	49	0.5	Logistic regression	USG	1857	1783	435	361
Ji et al.^35^	2022	China	304,145	62	0.3933	Random forest	USG	63,950	65,729	9811	7804
Qin et al.^36^	2023	China	4332	48.4	0.766	Random forest	USG	1649	2687	271	272
Peng et al.^37^	2023	China	709	37		Random forest	MRI	88	26	9	8
Lee et al.^38^	2023	USA	610	52	0.5	CNN	Liver Biopsy	-			
Razmpour et al.^39^	2023	Iran	513	37	0.53	Random forest	USG	-			
Yaghouti et al.^40^	2024	Iran	181			Random forest	Clinical data	107	40	17	17
Qi et al.^41^	2024	China	208	54	0.586	Logistic regression	Liver Biopsy	-			

### Risk of bias assessment

**Fig. 2:**
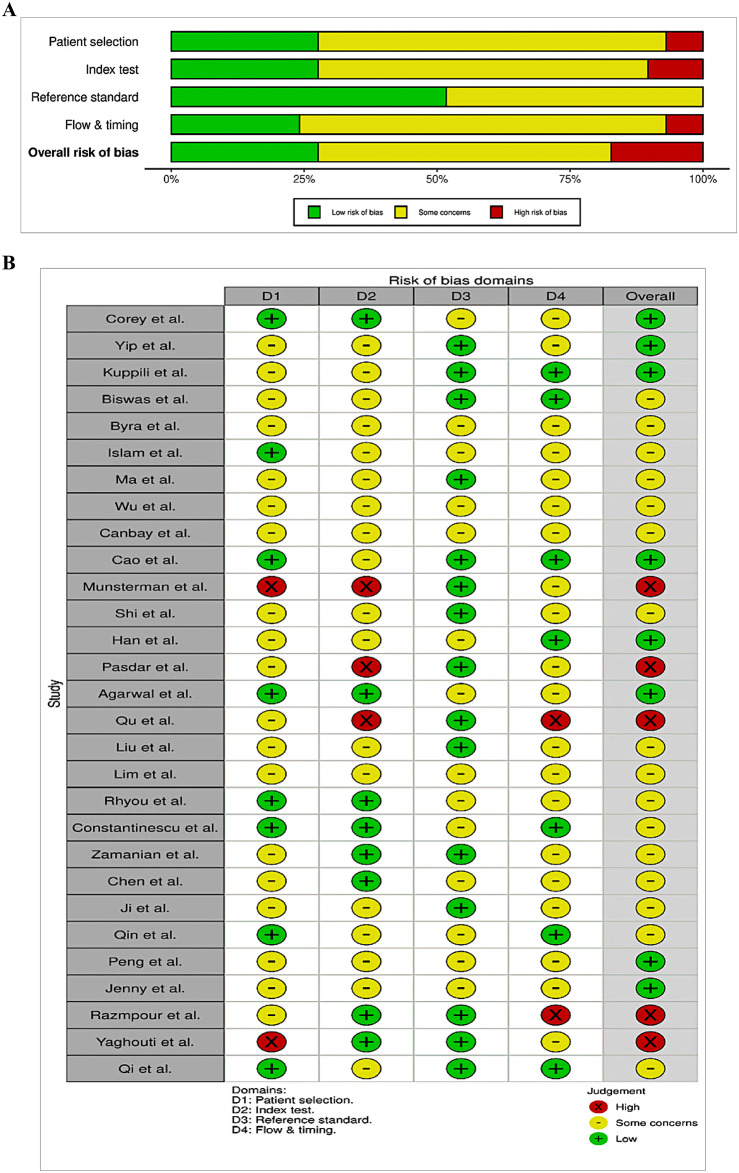
(**A**) Summary graph of the quality assessment via the QUASAD-2 tool. (**B**) Domain wise quality assessment via the QUADAS-2 tool.

The methodological quality of all 29 studies included in the systematic review was evaluated via the QUADAS-2 tool, as summarized in Fig. 2A and B. Importantly, no study was rated as having a low risk of bias across all four assessed domains, reflecting a moderate to substantial risk across the evidence base. In the patient selection domain, 21% of the studies were rated as high risk, primarily due to the use of nonrandom or retrospective sampling or a lack of clarity regarding the inclusion criteria. Approximately 48% of the studies were classified as having some concerns, often due to incomplete reporting on recruitment methods, whereas only 31% were assessed as low risk.

The index test domain exhibited the highest uncertainty, with 55% of the studies categorized as having an unclear risk of bias. This was largely attributed to insufficient information about blinding to reference standards and a lack of predefined thresholds for AI algorithm outputs. An additional 14% of the studies were rated as high risk in this domain because of post hoc model calibration or insufficient internal validation. In the reference standard domain of the QUADAS assessment, only 10% of studies were rated as high risk. However, nearly half (48%) were flagged as having some concerns, primarily due to insufficient information about how the reference standard for NAFLD was verified or whether it was interpreted independently of the index test. Although some studies employed liver biopsy or MRI as reference standards, critical details such as whether pathologists or radiologists were blinded to the AI outputs were often inadequately reported. Notably, seven studies relied solely on imaging-based reference standards, without histological or biochemical confirmation, and were therefore classified as high risk in this domain. Studies that used ultrasound as both index and reference tests were retained but classified as ‘ultrasoundonly reference’ and scored high risk in the QUADAS referencestandard domain owing to potential diagnostic circularity.

The flow and timing domain also reflected inconsistencies, with 24% of studies showing a high risk of bias. Common reasons included a lack of uniform timing between the index test and reference standard or patient attrition without adequate explanation. Nearly half of the studies (45%) had some concerns in this domain. Overall, 55% of the included studies had at least one domain rated as either high risk or having some concerns, indicating notable heterogeneity in study conduct and reporting. Applicability concerns were generally low across all domains. However, 24% of the studies exhibited unclear applicability in the index test domain, stemming from vague or insufficient reporting of AI model characteristics, training data, or clinical input features.

### Quantitative synthesis

#### Publication bias

**Fig. 3:**
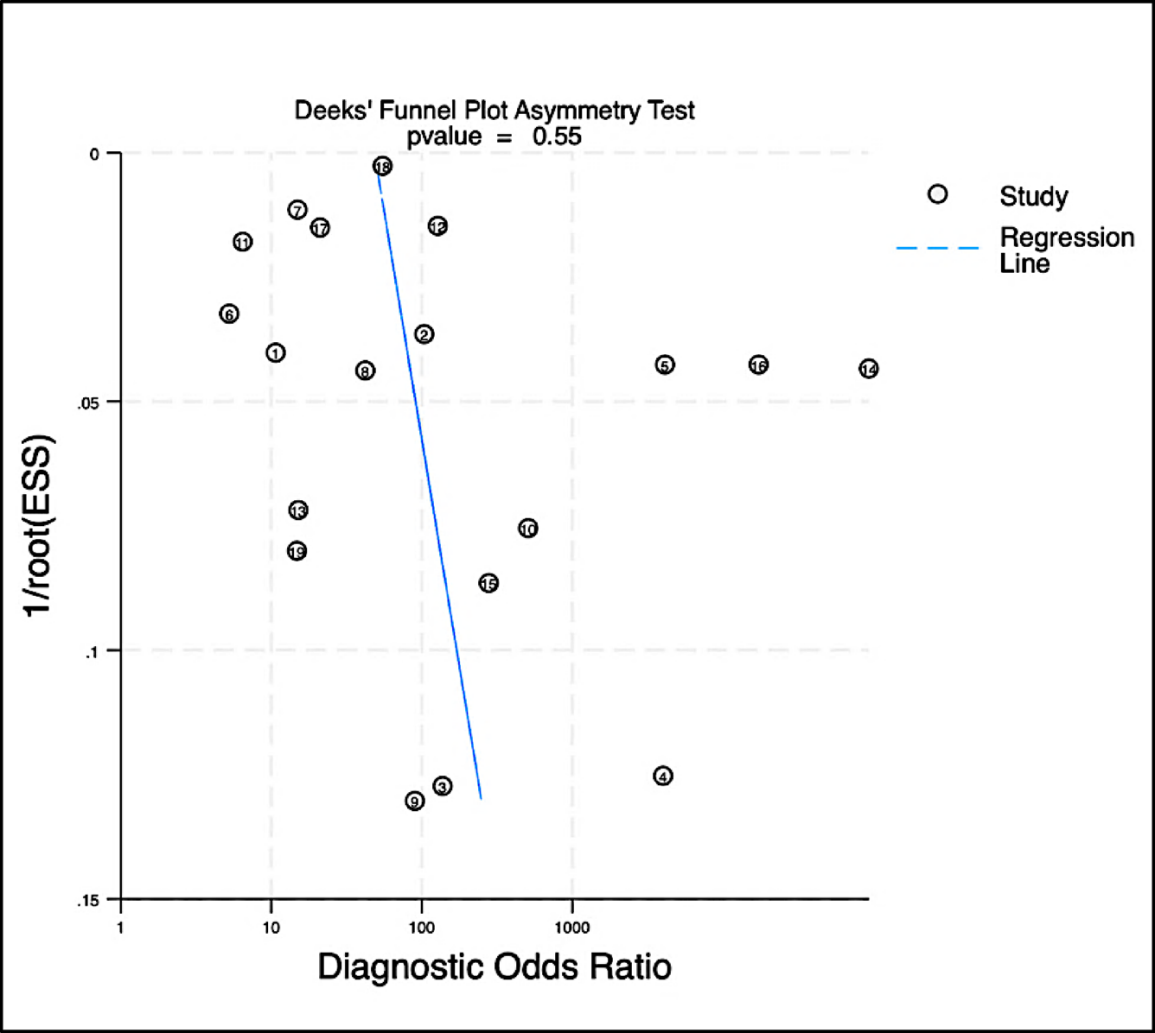
Deek’s funnel plot asymmetry test.

Publication bias was assessed via Deeks’ funnel plot (Fig. 3) asymmetry test for the 19 studies included in the meta-analysis. The regression slope was not statistically significant (*p* = 0.55), and the funnel plot was visually symmetrical, suggesting no evidence of small-study effects.

### Performance of AI in diagnosing NAFLD

#### Pooled sensitivity and specificity

**Fig. 4:**
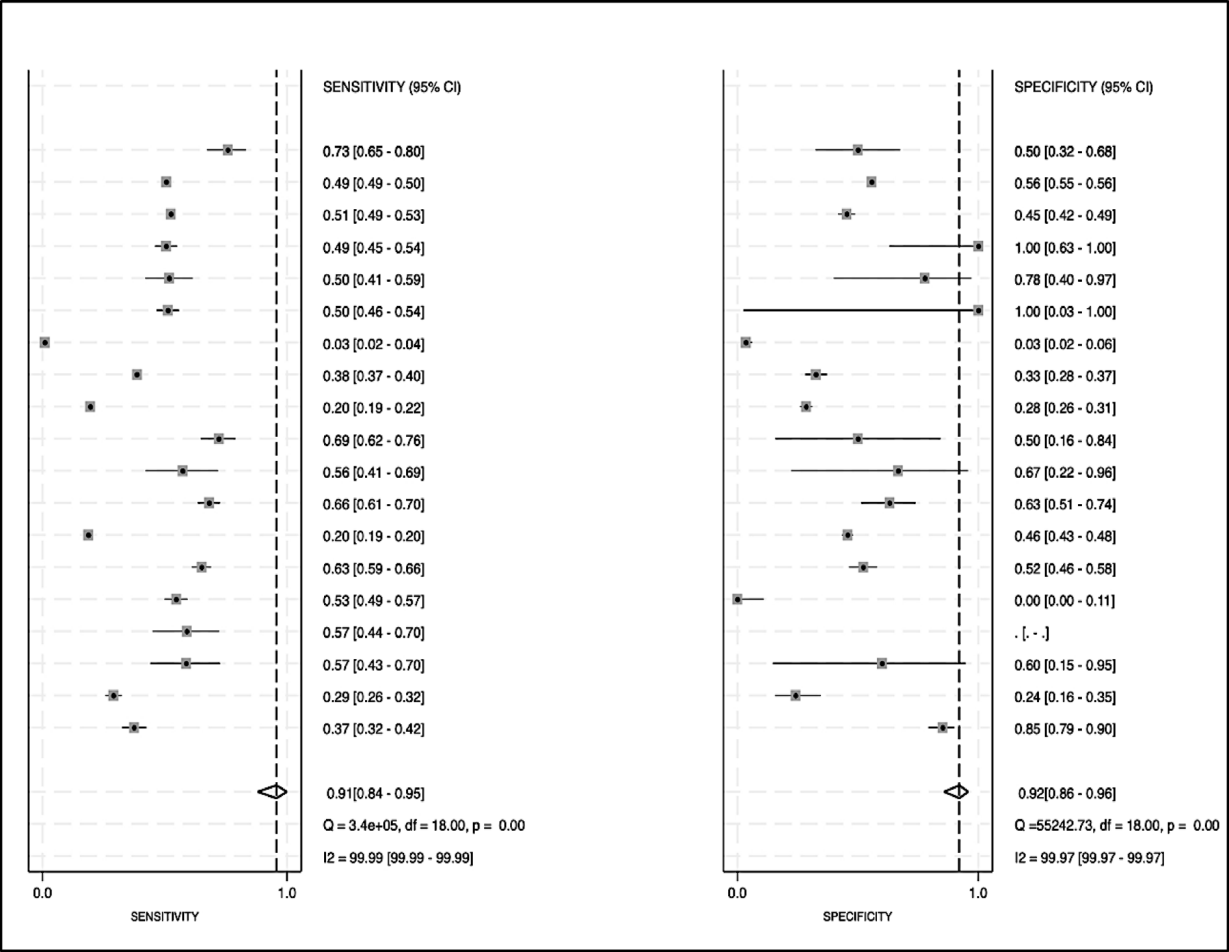
Forest plots of pooled sensitivity and specificity.

Of the 29 studies included in the systematic review, 19 provided sufficient 2 × 2 data to be included in the meta-analysis. The forest plots (Fig. 4) illustrate the individual and pooled estimates of sensitivity and specificity across the 19 included studies. The pooled sensitivity of the AI models for detecting NAFLD was 91% (95% CI: 84–95%). Similarly, the pooled specificity was 92% (95% CI: 86–96%), suggesting a high ability of the AI to correctly exclude individuals without NAFLD. Heterogeneity was quantitatively confirmed, with an I² statistic of 99.99% for sensitivity and 99.97% for specificity, both of which were accompanied by statistically significant Q tests (*p* < 0.001), supporting the use of a random effects model. The bivariate model parameters are given in Table S3. A smaller subset of studies employed clinical variables and these models differed widely in their variable sets and validation strategies and hence we did not pool their accuracy.

### Summary receiver operating characteristic (HSROC) and AUC

**Fig. 5:**
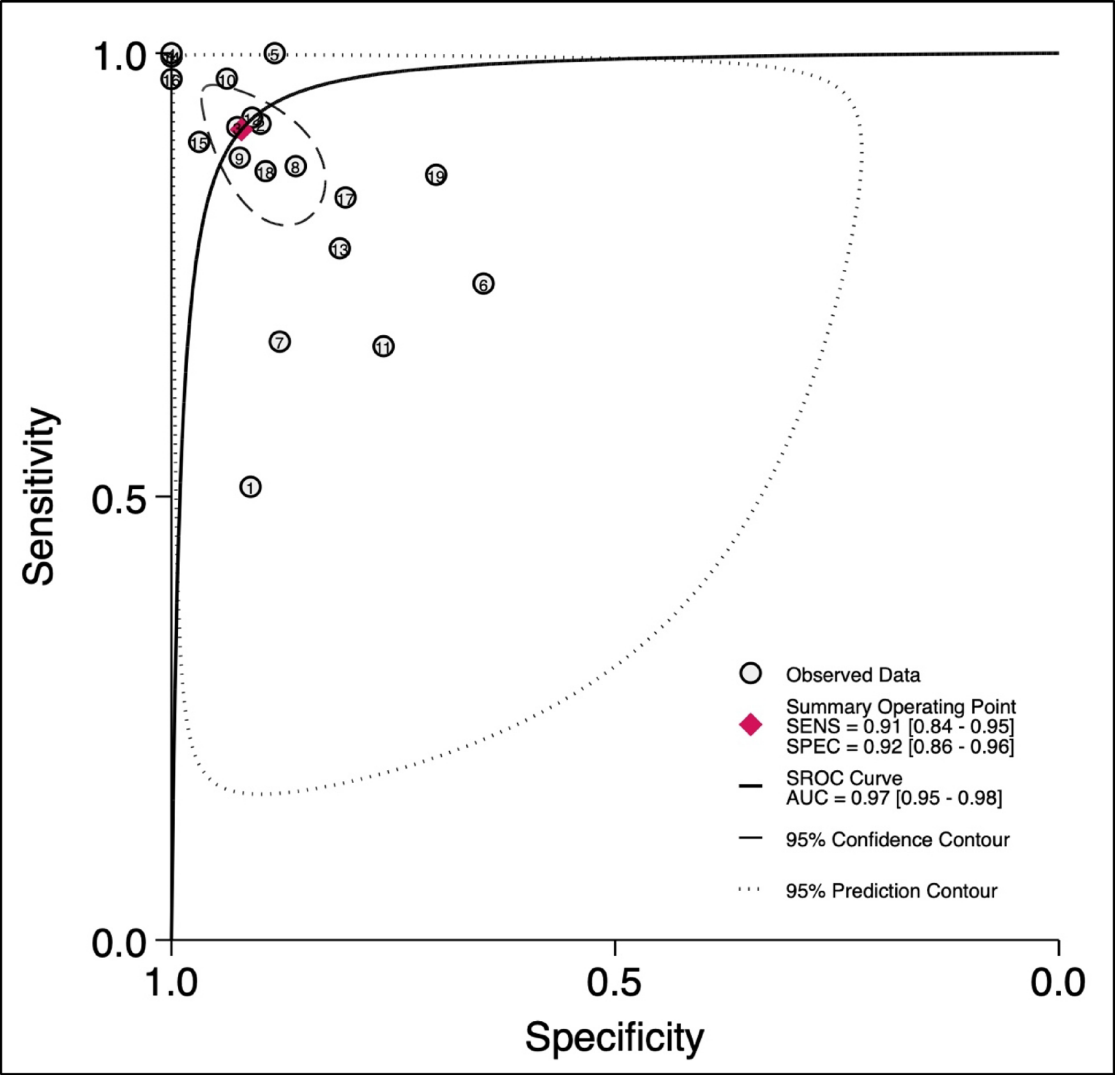
Hierarchical summary receiver operating characteristic (HSROC) curve.

The HSROC curve (Fig. 5) summarizes the overall diagnostic performance of AI-based models in detecting NAFLD. The summary operating point reflects a pooled sensitivity of 0.91 (95% CI: 0.84–0.95) and a pooled specificity of 0.92 (95% CI: 0.86–0.96). The area under the curve (AUC) was 0.97 (95% CI: 0.95–0.98), demonstrating excellent overall diagnostic accuracy. The clustering of most observed data points near the top-left corner of the plot further reinforces the strong diagnostic performance of the AI models. The HSROC model parameters are given in Table S4.

#### Diagnostic odds ratio (DOR)

The pooled diagnostic odds ratio (DOR) for AI-based models in diagnosing nonalcoholic fatty liver disease (NAFLD) was 123.7 (95% CI: 76.2–412.5), underscoring the strong overall discriminatory performance of these models. The DOR synthesizes sensitivity and specificity into a single metric, indicating that AI tools are more than 100 times more likely to correctly differentiate between individuals with and without NAFLD.

#### Fagan nomogram and posttest probabilities

**Fig. 6:**
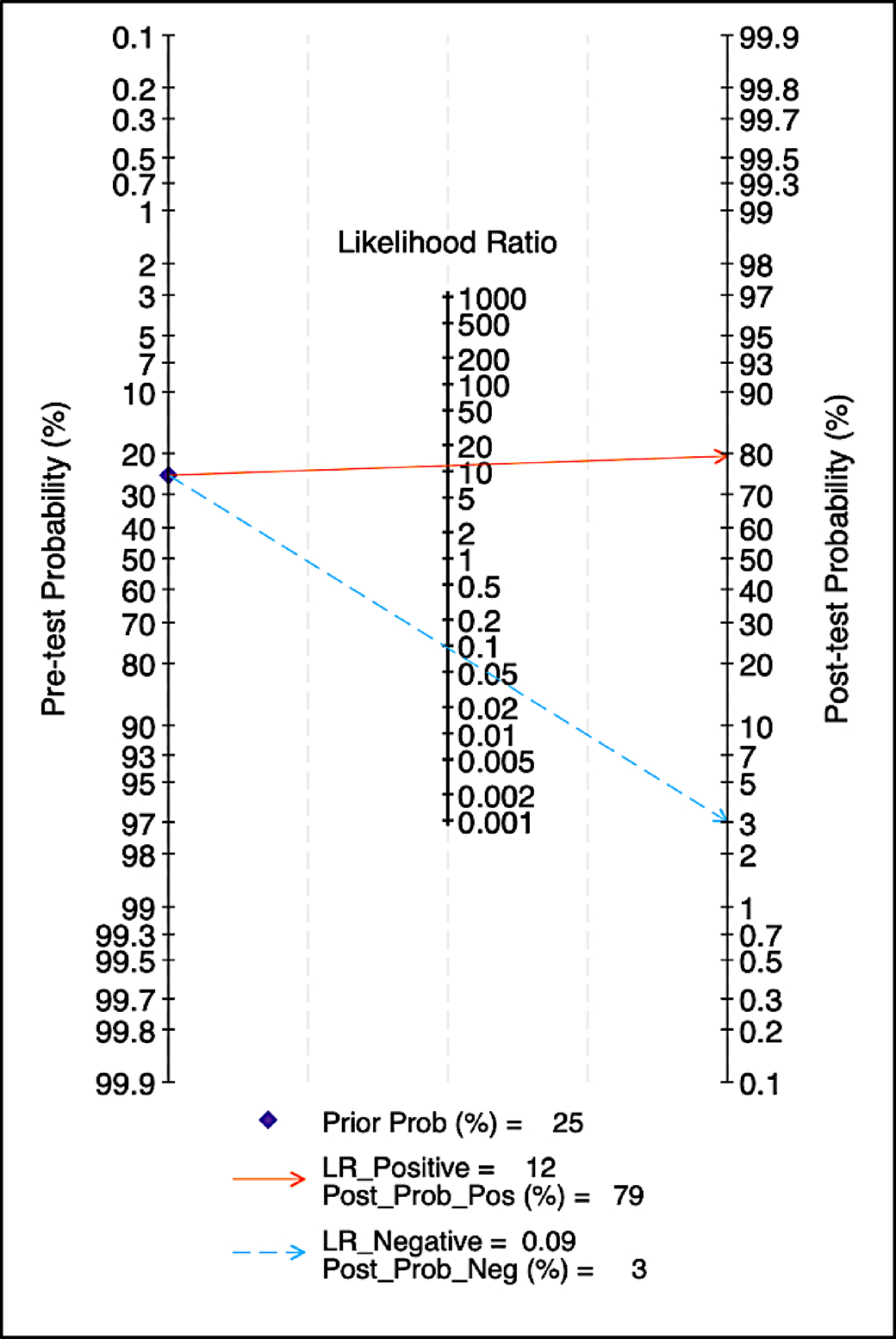
Fagan’s Nomogram Demonstrating Posttest Probabilities Based on Pretests.

Assuming a pretest probability of 25%, which is reflective of real-world prevalence in at-risk or asymptomatic populations, a positive test result increased the posttest probability of nonalcoholic fatty liver disease (NAFLD) to 79%, corresponding to a pooled positive likelihood ratio (LR⁺) of 12. Conversely, a negative result reduced the posttest probability to 3%, corresponding to a pooled negative likelihood ratio (LR⁻) of 0.09 (Fig. 6).

**Fig. 7:**
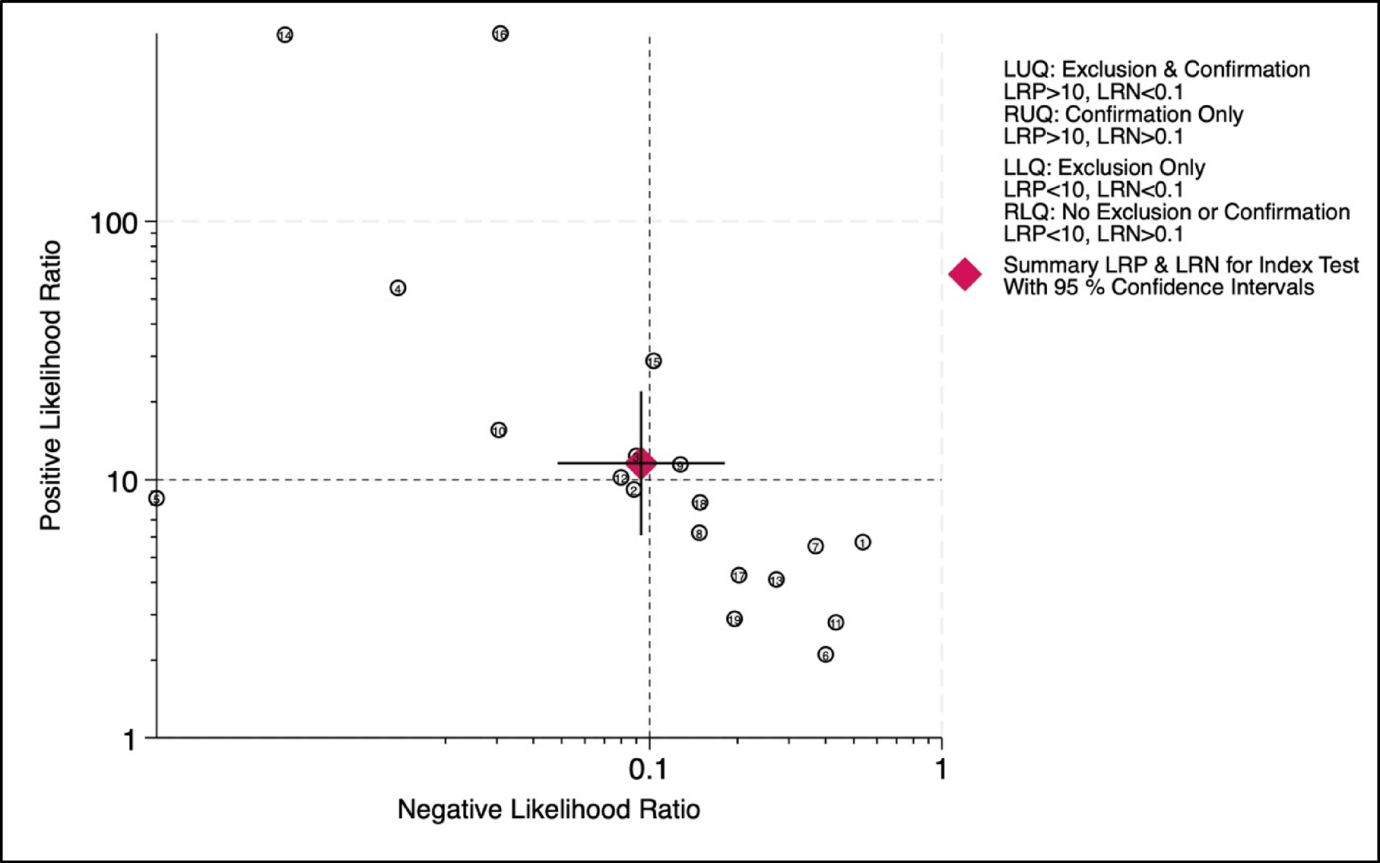
Likelihood ratio scatter plot of individual studies showing diagnostic accuracy with summary points and 95% confidence intervals.

### Probability and likelihood ratio

#### Summary of likelihood ratio analysis

The LR scatter plot (Fig. 7) depicts the distribution of individual studies across predefined diagnostic thresholds for exclusion (LR⁻ <0.1) and confirmation (LR⁺ >10). The majority of studies clustered in the upper left quadrant, indicating strong diagnostic performance for both ruling in and ruling out NAFLD. The summary point, marked by a red diamond with 95% confidence intervals, also lies within this quadrant, further supporting the overall high diagnostic utility of the index test.

#### Posterior probability and predictive value analysis

**Fig. 8:**
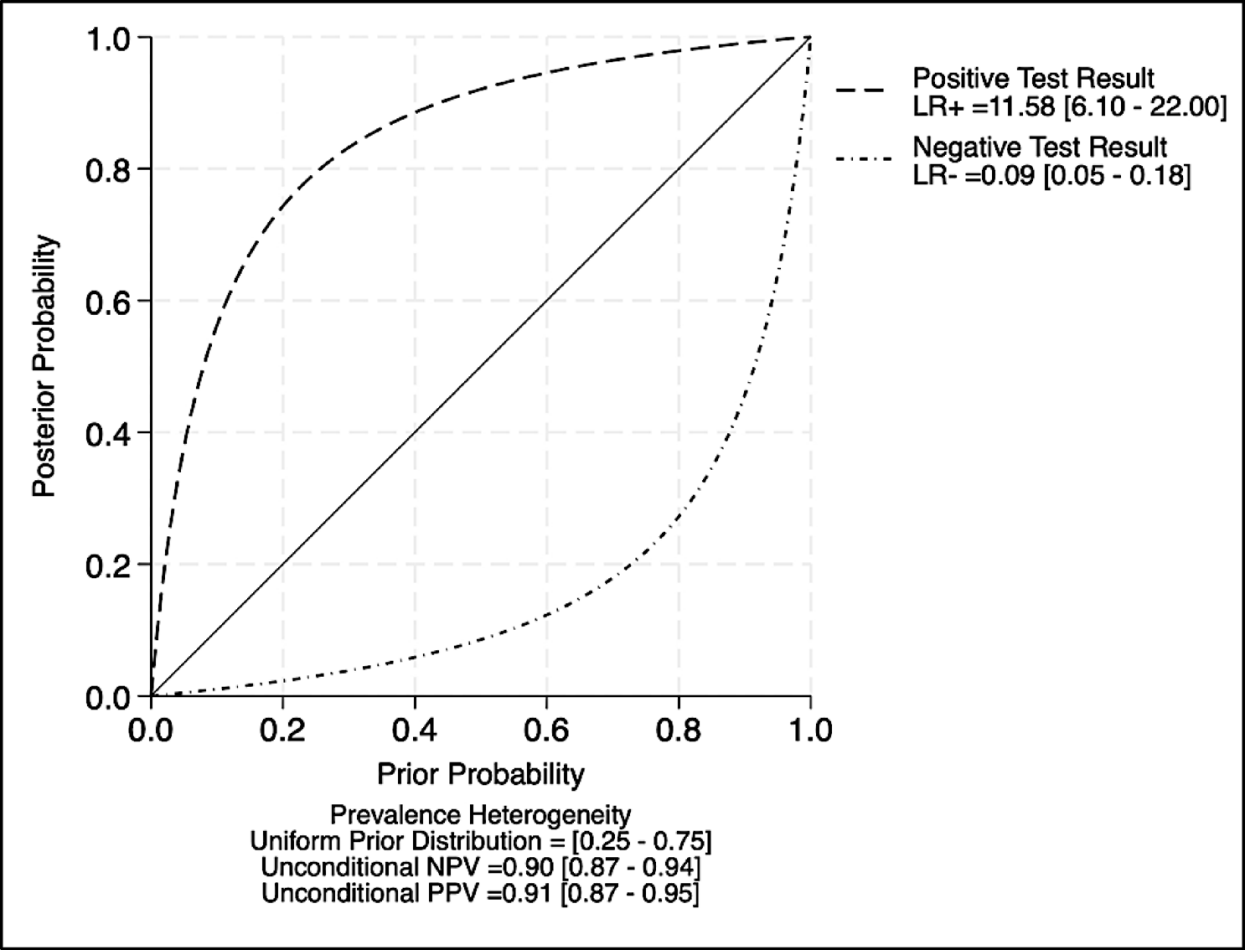
Posterior probability curve illustrating the impact of pretest probability and likelihood ratios on diagnostic test interpretation.

Posterior probability curves (Fig. 8) illustrate how varying prior probabilities influence diagnostic certainty following a positive or negative test result. Across a uniform prior range of 0.25–0.75, the test demonstrated strong separation, with a summary LR⁺ of 11.58 (95% CI: 6.10–22.00) and an LR⁻ of 0.09 (95% CI: 0.05–0.18). The corresponding pooled unconditional positive predictive value (PPV) was 0.91 (95% CI: 0.87–0.95), and the negative predictive value (NPV) was 0.90 (95% CI: 0.87–0.94), confirming the high discriminatory ability and reliability of the AI-based diagnostic tool across varying disease prevalence contexts. The summary point estimates are given in Table S5.

#### Model diagnostics and statistical fit

**Fig. 9:**
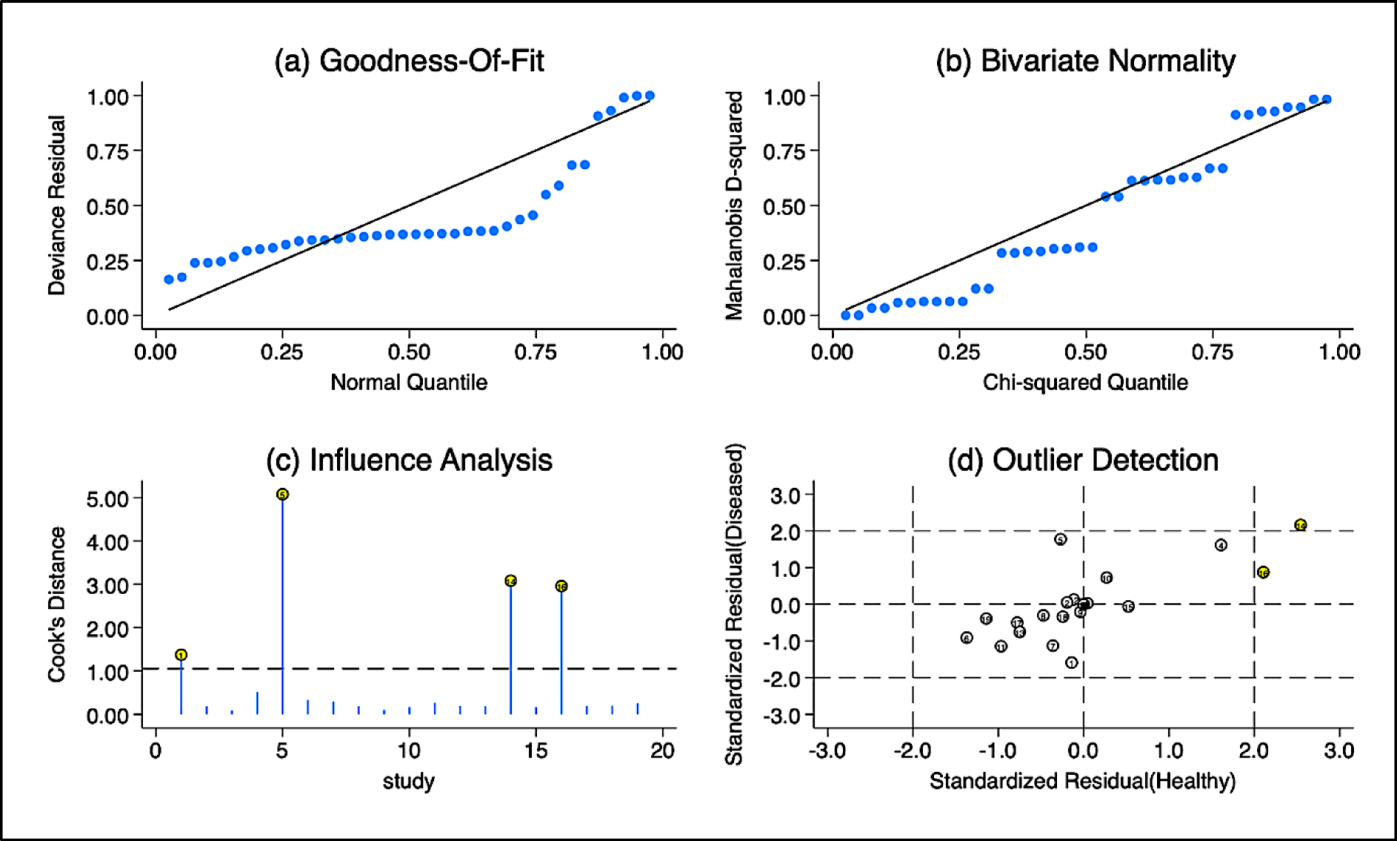
Diagnostic meta-regression model diagnosis-goodness-of-fit, bivariate normality, influence, and outlier analysis.

To evaluate the statistical robustness of the meta-analytic model, multiple diagnostic plots were generated as shown in Fig. 9. The goodness-of-fit plot (Fig. 9, panel a) showed minor deviations from the expected 45-degree line at the extremes, suggesting a slight departure from ideal residual normality. The Mahalanobis distance Q‒Q plot (Fig. 9, panel b) indicates general adherence to bivariate normality assumptions, with most studies falling along the theoretical line. Influence analysis (Fig. 9, panel c) using Cook’s distance identified four studies with values > 1, implying a notable impact on the overall model estimates. Furthermore, the outlier detection plot (Fig. 9, panel d) revealed a small number of data points with standardized residuals greater than ± 2, suggesting the presence of influential outliers that may have contributed to between-study heterogeneity. Nonetheless, the overall model stability was preserved, with minimal distortion in pooled estimates upon inspection.

### Subgroup analysis and sensitivity analysis

The subgroup analysis revealed the differential diagnostic performance of the AI models on the basis of classifier type, NAFLD status, reference standard, and validation method, as shown in Table 2. Among classifiers, convolutional neural networks (CNNs) exhibited the highest accuracy, with a sensitivity of 0.98, specificity of 0.97, and perfect AUC of 1.00, outperforming logistic regression (AUC: 0.98) and random forest (AUC: 0.90). Diagnostic performance was marginally greater in studies using suspected NAFLD cases (AUC: 0.92) than in those using confirmed cases (AUC: 0.89). When stratified by the reference standard, liver biopsy, although considered the gold standard, yielded slightly lower sensitivity (0.782) and AUC (0.913) than did imaging-based standards such as ultrasound (AUC: 0.918) and MRI (AUC: 0.89). The validation method also influenced accuracy, with generic cross-validation (AUC: 0.927) and 10-fold cross-validation (AUC: 0.906) outperforming the external validation (AUC: 0.91) and 5-fold methods (AUC: 0.898). After excluding small studies, the pooled sensitivity and specificity both remained high at 0.88, with a preserved AUC of 0.91. Exclusion of eight studies with < 500 participants yielded comparable accuracy (sensitivity 0.86, 95% CI 0.77–0.92; specificity 0.89, 95% CI 0.82–0.93) and similar heterogeneity (I² 78%), indicating that very small cohorts did not influence the pooled effect (Figure S1 A and B). When restricted to nine studies that used MRI‑PDFF or liver biopsy as the reference standard, pooled sensitivity and specificity were 0.88 (95% CI 0.80–0.93) and 0.93 (0.86–0.96), yielding an AUC of 0.94 comparable to, and slightly higher than, the ultrasound‑referenced subgroup (AUC 0.92). On the basis of the influence analysis, we found that studies by Corey et al., Byra et al., Rhyou et al., and Zamanian et al. were outliers; hence, after removing these 4 studies, we obtained sensitivity and specificity values of 88% (95% CI: 82–92%) and 88% (95% CI: 83–92%), respectively.

**Table 2:** Subgroup analysis of diagnostic performance metrics of AI models for NAFLD detection.

Subgroup Variable	Category	Study Count	SN	SP	AUC
AI Classifier	CNN	4	0.98	0.97	1.00
	Logistic regression	9	0.91	0.94	0.98
	Random forest	6	0.84	0.82	0.90
NAFLD Status	Confirmed	8	0.824	0.88	0.89
	Suspected	11	0.858	0.869	0.92
Reference Standard	Liver Biopsy	5	0.782	0.902	0.913
	MRI	3	0.853	0.86	0.89
	USG	10	0.87	0.881	0.918
Validation Method	10-fold cross-validation	9	0.851	0.903	0.906
	5-fold cross-validation	2	0.835	0.811	0.898
	Cross-validation	5	0.875	0.865	0.927
	External validation	2	0.732	0.916	0.91

## Discussion

### Summary of the study findings

This systematic review appraises AI algorithms derived from both imaging and nonimaging (clinical) data sources for detecting hepatic steatosis synthesizing evidence from 29 peer-reviewed studies published between 2013 and 2024. Among these studies, 19 provided sufficient data for meta-analysis. The pooled sensitivity and specificity of the AI models were 91% and 92%, respectively, with an area under the curve (AUC) of 0.97, indicating excellent diagnostic performance. Convolutional neural networks (CNNs) and models validated against magnetic resonance imaging (MRI) or liver biopsy demonstrated superior performance. The diagnostic odds ratio (DOR) was 123.7, suggesting that AI-based tools are highly effective at distinguishing between NAFLD and non-NAFLD individuals. Fagan’s nomogram analysis revealed that AI testing substantially altered posttest probabilities, confirming its clinical utility in both high- and low-prevalence settings.

Subgroup analyses highlighted the impact of the AI architecture, reference standards, and validation strategies on diagnostic outcomes. The externally validated models showed slightly lower performance but offered greater generalizability. Despite the overall robust findings, high between-study heterogeneity was observed. Beyond classifier type and reference standard, heterogeneity likely stems from poorly reported study details. Most papers gave only mean age without agestratified or metabolicrisk breakdowns; imaging parameters (e.g., MRI field strength, ultrasound presets) and key modeltraining steps (dataset size after filtering, augmentation, hyperparameters, external validation) were seldom specified. The risk of bias assessment revealed moderate quality across studies, with particular concerns in the index test and flow domains. Nevertheless, this review underscores the potential of AI, particularly deep learning models, to support noninvasive, cost-effective, and scalable NAFLD diagnosis. Further prospective, multicentric studies with standardized AI reporting and external validation are needed to confirm these findings and inform clinical adoption.

We acknowledge the recent shift in terminology from NAFLD to Metabolic dysfunction–Associated Steatotic Liver Disease (MASLD), which better reflects the central role of metabolic risk factors in hepatic steatosis. However, our meta-analysis was based on studies published prior to the widespread adoption of the MASLD framework in 2023, during which the term NAFLD remained the prevailing diagnostic label. Importantly, there is substantial overlap between the two definitions, as the vast majority of individuals previously classified under NAFLD would also meet MASLD criteria due to common underlying metabolic dysfunction. Therefore, the diagnostic and prognostic insights derived from NAFLD-labeled cohorts remain relevant and translatable to current clinical practice. We have also clarified this point in the revised manuscript to reflect the evolving nomenclature and its implications.

### Comparison with existing literature

Our findings demonstrate that AI models achieve consistently high diagnostic accuracy for NAFLD, which aligns closely with results from prior meta-analyses. In this review of 19 studies, the pooled sensitivity (91%) and specificity (92%) were nearly identical to those reported in a 2023 meta-analysis, which reported pooled sensitivity and specificity values of 92% (90–93%) and 94% (93–96%), respectively^42^. That study also reported a diagnostic odds ratio (DOR) of 182 and an AUC of 0.98, closely matching our pooled DOR of 119.6 and an AUC of 0.97. Minor variations may reflect differences in study selection or recent literature updates, but the overall trend underscores the reproducibility of AI diagnostic performance. Notably, both meta-analyses suggest a clear advantage of AI-assisted models over conventional interpretation. For example, a landmark 2011 meta-analysis reported that human-interpreted ultrasound sensitivity was approximately 85% for moderate steatosis^43^, whereas AI-enhanced imaging in more recent studies achieved sensitivities exceeding 90%^44^. This improvement highlights the technological progress and enhanced detection capability of modern AI algorithms.

The superior performance of AI, particularly deep learning convolutional neural networks (CNNs), is grounded in their ability to identify subtle, pixel-level variations across entire liver images that may be imperceptible to human readers. CNNs integrate diffuse imaging features into a cohesive diagnostic signal, reducing random error and eliminating inter-operator variability. These strengths are reflected in our observed sensitivity range (~ 91–97%) and near-perfect AUCs. The diagnostic consistency of AI, in contrast to operator-dependent ultrasound, likely accounts for its superiority in both our findings and prior literature^43^. The high pooled DOR (~ 120), LR⁺ (~ 12), and LR⁻ (~ 0.09) further support the strong discriminatory capacity of the AI.

Subgroup analyses also revealed that AI models evaluated against imaging-based reference standards such as MRI-derived fat fraction or expert-graded ultrasound demonstrated higher diagnostic accuracy than those benchmarked against liver biopsy. This is consistent with findings from previous meta-analyses, where AI performance against ultrasound yielded specificities of ~ 97%, whereas it was ~ 88% when histology was used as the gold standard¹².

Percutaneous liver biopsy is the reference standard for grading hepatic steatosis; nevertheless, a core specimen represents < 1/50 000 of the liver and paired‑sample studies report histological discordance rates of 25–41%^45^, chiefly because steatosis is heterogeneously distributed within the parenchyma^46^.

AI systems, which analyze the entire liver image, may accurately detect fat accumulation overlooked by biopsy, thereby resulting in false positives relative to an imperfect gold standard. In contrast, full-liver imaging offers a more comprehensive and aligned reference, enhancing the synergy between AI learning and the ground truth. Thus, the diagnostic robustness of AI for NAFLD is not only reflected in statistical metrics but also supported by theoretical and methodological congruence with its imaging-based foundations. Several factors likely contributed to the heterogeneity observed across studies. Patient characteristics differed considerably, with mean ages ranging from 42 to 57 years and BMI values between 25 and 33 kg/m², along with varying prevalence of metabolic comorbidities; all of which can influence steatosis thresholds. Imaging protocols were similarly inconsistent: ultrasound studies employed transducers ranging from 3 to 7 MHz with heterogeneous machine settings; CT studies used attenuation cut-offs from 40 to 60 HU; and MRI sequences varied in terms of technique and field strength, each of which may affect image quality and diagnostic accuracy. In terms of AI model development, there was substantial variation some studies utilized large, multicenter datasets with detailed cross-validation, while others were based on smaller, single-center cohorts with limited information regarding preprocessing methods, data augmentation strategies, or hyperparameter optimization. We revisited all included studies to further investigate these sources of variation but found that reporting was frequently sparse and inconsistent. Most studies provided only basic demographic data, and essential details on imaging protocols and model development were often missing, which limited our ability to conduct additional subgroup analyses or meta-regression.

Compared with Zhao et al. (2023)^42^, a meta-analysis conducted with similar objectives, our update offers four advances. First, it incorporates 14 additional cohorts, including several multi‑centre deep‑learning investigations published after January 2023, thereby reflecting the current AI landscape. Second, we present the first head‑to‑head pooled comparison of conventional machine‑learning and deep‑learning models, which reveals near‑identical sensitivity (90% vs. 91%) and specificity (both 92%) but highlights the superior AUC of contemporary CNN variants (1.00) over random forests (0.90) and logistic regression (0.98). Third, by separating internally cross‑validated models (AUC 0.927) from externally validated models (AUC 0.91) we quantify the modest performance drop that the earlier review which combined designs could not detect. Finally, we translate statistical accuracy into bedside decision support through likelihood‑ratio scatter plots and Fagan nomograms at a 25% pre‑test prevalence, showing post‑test probabilities of 79% for positive and 3% for negative results.

Although imagingbased algorithms dominate current research, a few investigators have shown that models trained solely on routinely collected clinical information such as anthropometric indices, serum lipids, glucoseinsulin markers, and liver enzymes can reliably flag individuals with hepatic fat accumulation. Large cohort and biobank analyses^47^ indicate that these variable sets, when processed through decisiontree ensembles or gradientboosting frameworks, discriminate steatosis at a level approaching that of imagebased tools, making them attractive as firstline triage tests in primarycare or resourcelimited settings^48^. By exploiting data already captured in routine consultations, these algorithms could triage candidates for confirmatory imaging or biopsy. Yet heterogeneity in variable choice, preprocessing, and study populations persists, so robust external validation against quantitative imaging or histology is still required before clinical rollout^49^.

### Clinical and public health implications

The findings of this meta-analysis highlight the substantial clinical utility of artificial intelligence (AI) in the noninvasive diagnosis of nonalcoholic fatty liver disease (NAFLD) through the estimation of hepatic steatosis. With a pooled sensitivity and specificity exceeding 90% and an HSROC AUC of 0.97, AI, particularly deep learning convolutional neural networks (CNNs), applied to imaging data demonstrate diagnostic performance comparable to that of invasive gold standards such as liver biopsy. In clinical settings, these results suggest that AI-based models can serve as reliable decision-support tools, augmenting existing diagnostic workflows and enabling earlier identification of NAFLD. This is especially pertinent in resource-limited environments, where access to advanced modalities such as MRI-PDFF or histopathological confirmation is often restricted. By leveraging routine data sources such as ultrasound or clinical parameters, AI-enabled tools can facilitate timely risk stratification and intervention, potentially mitigating the progression to advanced fibrosis or cirrhosis.

From a public health standpoint, the implementation of scalable, low-cost AI-driven diagnostic solutions offers a pragmatic approach to tackling the growing global burden of NAFLD, particularly in low- and middle-income countries (LMICs). As NAFLD now affects an estimated 25–30% of the global population^50^, with the prevalence increasing in parallel with obesity and diabetes, the need for efficient population-level screening has become urgent. AI algorithms, once externally validated and integrated into health systems, can support the WHO’s Global Strategy on Digital Health 2020–2025 by enhancing diagnostic reach and equity in underserved settings^51^. Furthermore, the high negative predictive value observed in this analysis underscores AI’s utility in effectively ruling out disease, thereby reducing unnecessary referrals and invasive investigations, conserving healthcare resources, and improving triage efficiency. In alignment with Sustainable Development Goals, which target universal health coverage and reduce noncommunicable disease burden, the adoption of AI in NAFLD diagnostics represents a timely innovation with meaningful implications for liver health surveillance and chronic disease management on a global scale^50^.

### Strengths and limitations

This systematic review and meta-analysis have several notable strengths. It is one of the few focused evaluations of artificial intelligence (AI) models for diagnosing nonalcoholic fatty liver disease (NAFLD), synthesizing data from 29 studies and quantitatively pooling results from 19 eligible articles. The study adheres to the PRISMA 2020 and Cochrane Handbook guidelines, ensuring methodological rigor. Restricting the search to the past decade has enhanced clinical relevance, reflecting current AI developments. Diverse AI models, including convolutional neural networks, random forests, and logistic regression, were assessed across multiple reference standards, such as ultrasound, MRI, and liver biopsy. This diversity allowed robust subgroup analyses and improved generalizability. The application of hierarchical summary receiver operating characteristic (HSROC) modeling, diagnostic odds ratios (DORs), and Fagan’s nomogram enhanced the interpretability of diagnostic accuracy. Additionally, the use of QUADAS-2 for risk of bias assessment and sensitivity analyses reinforced the reliability of the findings. Nonetheless, some limitations must be acknowledged. First, our pooled estimates reflect the ability of AI algorithms to detect hepatic steatosis on imaging; most included studies did not systematically capture metabolicrisk profiles or alternative liver etiologies, and several used imagingonly reference standards. Consequently, the reported accuracy may overstate realworld performance for establishing a full MASLD diagnosis, which requires clinical and laboratory correlation. Considerable heterogeneity was observed across studies in terms of AI architecture, imaging protocols, reference standards, and study populations, which may have influenced pooled estimates. Although subgroup and sensitivity analyses were conducted, residual heterogeneity remained high, as reflected in the I² values for sensitivity and specificity. Several primary studies enrolled relatively small cohorts that heightens the risk of over‑fitting particularly for deep‑learning models. Nevertheless, sensitivity analysis that removed these studies produced virtually unchanged summary estimates, suggesting that the meta‑analytic conclusions are robust. A further limitation is that ten studies employed ultrasound as both the input modality and reference standard, introducing diagnostic circularity. Although such designs risk mere replication of human reads, our subgroup analysis demonstrated that models tested against MRI‑PDFF or biopsy performed at least as well, suggesting genuine incremental value. Furthermore, not all studies reported complete 2 × 2 diagnostic contingency data, resulting in the exclusion of several studies and the potential for selection bias. Sparse reporting of population descriptors, imaging acquisition parameters, and model-training details prevented multivariable meta-regression (minimum ≈ 10 studies per covariate) and leaves residual heterogeneity unexplained. The predominance of retrospective studies and the lack of external validation in most of the included studies may limit the generalizability of the results.

## Conclusion

This systematic review and meta-analysis confirm that artificial intelligence (AI) models, particularly those employing deep learning architectures such as convolutional neural networks, demonstrate high diagnostic accuracy for nonalcoholic fatty liver disease (NAFLD) through estimation of hepatic steatosis, with pooled sensitivity and specificity exceeding 90% and an area under the HSROC curve of 0.97. These findings support the integration of AI as a reliable, noninvasive adjunct to conventional diagnostic approaches, particularly in resource-limited settings where access to liver biopsy or advanced imaging is constrained. As the global prevalence of NAFLD continues to rise, AI-assisted tools offer scalable and potentially cost-effective solutions for early detection and risk stratification. Future research should prioritize prospective, multicentric validation studies across diverse populations, alongside health economic evaluations, to assess feasibility and real-world applicability. Successful implementation will also require investment in digital infrastructure, ethical governance, and clinician training to ensure the responsible and equitable adoption of AI-based diagnostic systems in routine liver disease management.

## Supplementary Information

Below is the link to the electronic supplementary material.

Supplementary Material 1

## Data Availability

All data generated or analyzed during this study are available from the corresponding author upon reasonable request.
