# Supplementary material for: Diagnostic accuracy of artificial intelligence models for imaging detection of hepatic steatosis through systematic review and meta analysis
Source: Sci Rep. 2025 Oct 2;15:34408. doi: 10.1038/s41598-025-17386-3 (PMC12491402; doi:10.1038/s41598-025-17386-3)
Supplement: Supplementary file 1 — Supplementary Material 1 [file 41598_2025_17386_MOESM1_ESM.docx]

Table of Contents

[Table S1: PubMed search strategy 2](#_Toc204329676)

[Table S2: Characteristics of the studies included in the systematic review and meta-analysis 3](#_Toc204329677)

[Table S3: Bivariate Model Estimates 6](#_Toc204329678)

[Table S4: HSROC Model Parameters 7](#_Toc204329679)

[Table S5: Summary Point Estimates 8](#_Toc204329680)

[Figure S1 A and B: Sensitivity analysis for studies with less than 500 participants 9](#_Toc204329681)

# Table S1: PubMed search strategy

| **Blocks** | **Keywords** | **Records retrieved** |
| --- | --- | --- |
| I | "Algorithms” [MeSH Terms] OR "Artificial Intelligence" [MeSH Terms] OR "Machine Learning" [MeSH Terms] OR "Deep Learning" [MeSH Terms] OR "Support Vector Machine" [MeSH Terms] OR "Regression Analysis" [MeSH Terms] OR "Random Forest"[MeSH Terms] OR "Logistic Models" [MeSH Terms] OR "Neural Networks, Computer” [MeSH Terms] OR “computational intelligence” [tiab] OR “machine intelligence” [tiab] OR “Hierarchical Learning” [tiab] OR “Logit Model” [tiab] OR “least squares” [tiab] OR “Neural Network Model” [tiab] OR “Connectionist Model” [tiab] | 954,152 |
| D | "Non-alcoholic Fatty Liver Disease"[MeSH Terms] OR “NAFLD” [tiab] OR “Fatty Liver, Nonalcoholic” [tiab] OR “fatty liver” [MeSH Terms] OR “nonalcoholic steatohepatitis” [tiab] OR “NASH” [tiab] OR “steatohepatitis” [tiab] OR “liver steatosis” [tiab] OR “MAFLD” [tiab] OR “Metabolic-associated fatty liver disease” [tiab] OR “Metabolic dysfunction-associated fatty liver disease” [tiab] OR “Visceral Steatosis” [tiab] | 67,449 |
| R | "Sensitivity and specificity"[MeSH Terms] OR “sensitivity” [tiab] OR “specificity” [tiab] OR “accuracy” [tiab] OR “Diagnosis, Computer-Assisted"[MeSH Terms] OR “Computer-Assisted Diagnoses” [tiab] OR "Area Under Curve"[MeSH Terms] OR “AUC” [tiab] | 2,332,727 |
| P | 1 AND 2 AND 3 | 799 |

# Table S2: Characteristics of the studies included in the systematic review and meta-analysis

| **Author** | **Year of Publication** | **Country** | **Study Design** | **Study Population** | **Suspected/Confirmed NAFLD** | **Sample Size** | **Mean/Median Age** | **Proportion of Females (%)** | **Classifier** | **Training Data Set Size** | **Testing Data Set Size** | **Validation Method** | **Reference standard** | **TP** | **TN** | **FP** | **FN** |
| --- | --- | --- | --- | --- | --- | --- | --- | --- | --- | --- | --- | --- | --- | --- | --- | --- | --- |
| Corey et al. | 2016 | USA | Retrospective cohort | Adults from healthcare system | Confirmed | 620 | 61.6 | 0.56 | Logistic regression | 620 | 611 | External validation | Liver Biopsy | 161 | 276 | 27 | 155 |
| Yip et al. | 2017 | Hong Kong | Prospective cohort | General population | Confirmed | 922 | 48.1 | 0.57 | Logistic regression | 500 | 422 | 10-fold cross-validation | MRI | 243 | 592 | 66 | 21 |
| Kuppili et al. | 2017 | Portugal | Prospective cohort | Adults with USG-identified NAFLD | Suspected | 63 | 43 | 0.5 | Logistic regression | 63 |  | External validation | USG | 33 | 25 | 2 | 3 |
| Biswas et al. | 2018 | Portugal | Cross-sectional | Adults with USG-diagnosed NAFLD | Confirmed | 101 | 50 | 0.48 | Logistic regression | 70 | 31 | 10-fold cross validation | USG | 36 | 27 | 0 | 0 |
| Byra et al. | 2018 | Poland | Retrospective cohort | Obese patients undergoing bariatric surgery | Confirmed | 55 | 40.1 | 0.2 | CNN | 540 | 10 | 5-fold cross validation | Liver Biopsy | 38 | 15 | 3 | 0 |
| Islam et al. | 2018 | Taiwan | Retrospective cohort | Patients with FLD and controls | Suspected | 994 | 62 | 0.536 | Random forest | 794 | 200 | 10-fold cross-validation | USG | 439 | 260 | 141 | 154 |
| Ma et al. | 2018 | China | Cross-sectional | General population | Suspected | 10508 | 50 | 0.24 | Logistic regression |  |  | 10-fold cross-validation | USG | 1702 | 7012 | 974 | 820 |
| Wu et al. | 2018 | Taiwan | Retrospective cohort | Patients identified via health screening | Suspected | 577 | 54 | 0.549 | Random forest |  |  | 10-fold cross-validation | USG | 329 | 172 | 28 | 48 |
| Canbay et al. | 2019 | Germany | Retrospective & Prospective Cohort | Obese individuals (validation: bariatric surgery) | Confirmed | 286 | 43.5 | 0.76 | Logistic regression | 164 | 122 | 10-fold cross validation | Liver Biopsy |  |  |  |  |
| Cao et al. | 2019 | China | Prospective cohort | Obese adults | Confirmed | 240 | 41 | 0.45 | Logistic regression | 852 | 240 | Cross-validation | USG |  |  |  |  |
| Munsterman et al. | 2019 | Netherlands | Prospective cohort | Hospital-based population | Suspected/Confirmed | 79 |  |  | Random forest |  |  | Cross-validation | MRI |  |  |  |  |
| Shi et al. | 2019 | China | Retrospective cohort | Adults with confirmed NAFLD | Confirmed | 60 | 41 | 0.36 | Logistic regression | 248 | 100 | 10 fold Cross-validation | USG | 30 | 24 | 2 | 4 |
| Han et al. | 2020 | USA | Prospective cohort | Adults undergoing USG | Suspected | 204 | 52 | 0.6 | CNN | 102 | 102 | Cross-validation | MRI | 97 | 30 | 2 | 2 |
| Pasdar et al. | 2020 | UK | Prospective cohort | General population | Suspected | 1514 | 57 |  | Random forest | 1060 | 454 | 5-fold cross-validation | MRI | 677 | 2668 | 838 | 334 |
| Agarwal et al. | 2021 | USA | Cross-sectional | NAFLD patients (biopsy-proven) | Confirmed | 1016 | 48.3 | 0.615 | Random forest | 814 | 202 | 5-fold cross validation | Liver Biopsy |  |  |  |  |
| Qu et al. | 2021 | USA | Retrospective cohort | Pathology slides (NAFLD patients) | Suspected/Confirmed | 87 |  |  | Logistic regression | 65 | 22 | 4-fold cross-validation | Clinical data |  |  |  |  |
| Liu et al. | 2021 | China | Prospective cohort | Adults from hospital database | Confirmed | 3310 |  |  | Random forest | 10373 | 4942 | 10-fold cross-validation | Liver Biopsy | 1136 | 2802 | 280 | 724 |
| Lim et al. | 2021 | South Korea | Retrospective cohort | Living liver donors | Confirmed | 1652 | 31.4 | 0.337 | Logistic regression | 1165 | 487 | Cross-validation, internal validation | Liver Biopsy | 39 | 1298 | 304 | 11 |
| Rhyou et al. | 2021 | South Korea | Retrospective cohort | Adults undergoing USG for liver steatosis | Suspected | 3200 |  |  | Logistic regression | 1920 | 640 | 10 fold Cross-validation | USG | 1063 | 2133 | 0 | 3 |
| Constantinescu et al. | 2021 | Romania | Cross-sectional | General population | Suspected | 629 |  | 0.52 | CNN | 496 | 133 | Cross-validation | USG | 62 | 61 | 2 | 8 |
| Zamanian et al. | 2021 | Poland | Experimental study | Adults undergoing bariatric surgery | Suspected | 55 | 40 | 0.8 | CNN | 550 |  | 10-fold cross-validation | Liver Biopsy | 70 | 74 | 0 | 2 |
| Chen et al. | 2021 | China | Prospective cohort | Adults undergoing USG for NAFLD | Suspected | 7396 | 49 | 0.5 | Logistic regression | 5180 | 2216 | Cross-validation | USG | 1857 | 1783 | 435 | 361 |
| Ji et al. | 2022 | China | Retrospective cohort | Large-scale population | Suspected | 304145 | 62 | 0.3933 | Random forest |  |  | Cross-validation | USG | 63950 | 65729 | 9811 | 7804 |
| Qin et al. | 2023 | China | Retrospective cohort | Adults undergoing USG for NAFLD | Suspected | 4332 | 48.4 | 0.766 | Random forest | 10028 | 4411 | 10 fold Cross-validation | USG | 1649 | 2687 | 271 | 272 |
| Peng et al. | 2023 | China | Prospective cohort | General population undergoing USG | Suspected | 709 | 37 |  | Random forest | 578 | 131 | 10 fold Cross-validation | MRI | 88 | 26 | 9 | 8 |
| Jenny et al. | 2023 | USA | Cross-sectional | Biopsy-proven NAFLD patients | Confirmed | 610 | 52 | 0.5 | CNN | 742 | 242 | 10-fold cross-validation | Liver Biopsy |  |  |  |  |
| Razmpour et al. | 2023 | Iran | Prospective cohort | Obese individuals (BMI >30) | Suspected | 513 | 37 | 0.53 | Random forest |  |  |  | USG |  |  |  |  |
| Yaghouti et al. | 2024 | Iran | Retrospective cohort | NAFLD patients with clinical data | Confirmed | 181 |  |  | Random forest |  |  | Leave-one-out cross-validation (100 repetitions) | Clinical data | 107 | 40 | 17 | 17 |
| Qi et al. | 2024 | China | Retrospective cohort | MAFLD patients with biopsy-proven steatosis and controls | Confirmed | 208 | 54 | 0.586 | Logistic regression | 146 | 62 | 5-fold cross validation | Liver Biopsy |  |  |  |  |

# Table S3: Bivariate Model Estimates

| **Parameter** | **Coefficient** | **Std. Error** | **95% Confidence Interval** |
| --- | --- | --- | --- |
| E(logitSe) | 2.3613 | 0.3497 | 1.6759 to 3.0467 |
| E(logitSp) | 2.4573 | 0.3340 | 1.8024 to 3.1122 |
| Var(logitSe) | 2.0883 | 0.8900 | 0.9576 to 4.5544 |
| Var(logitSp) | 1.8032 | 0.8139 | 0.7445 to 4.3675 |
| Corr(logits) | 0.6888 | 0.1444 | 0.2980 to 0.8819 |

# Table S4: HSROC Model Parameters

| **Parameter** | **Coefficient** | **Standard Error** | **z-value** | **p-value** | **95% Confidence Interval** |
| --- | --- | --- | --- | --- | --- |
| Lambda | 4.8254 | 0.6132 | — | — | 3.6235 to 6.0272 |
| Theta | -0.1365 | 0.2859 | — | — | -0.6968 to 0.4239 |
| Beta | -0.0734 | 0.2486 | -0.30 | 0.768 | -0.5607 to 0.4139 |
| s2alpha | 6.5546 | 2.5664 | — | — | 3.0428 to 14.1196 |
| s2theta | 0.3019 | 0.1312 | — | — | 0.1288 to 0.7075 |

# Table S5: Summary Point Estimates

| **Measure** | **Estimate** | **Std. Err.** | **95% Confidence Interval** |
| --- | --- | --- | --- |
| Sensitivity (Se) | 0.9138265 | 0.0275379 | 0.8423586 to 0.9546387 |
| Specificity (Sp) | 0.9210944 | 0.0240861 | 0.8584385 to 0.9573949 |
| Diagnostic Odds Ratio (DOR) | 123.7903 | 76.02515 | 37.14715 to 412.5224 |
| Positive Likelihood Ratio (LR+) | 11.58127 | 3.788503 | 6.099698 to 21.98893 |
| Negative Likelihood Ratio (LR–) | 0.0935556 | 0.0314691 | 0.0483897 to 0.1808738 |
| Inverse LR (1/LR–) | 10.68883 | 3.595378 | 5.528581 to 20.6655 |

# Figure S1 A and B: Sensitivity analysis for studies with less than 500 participants


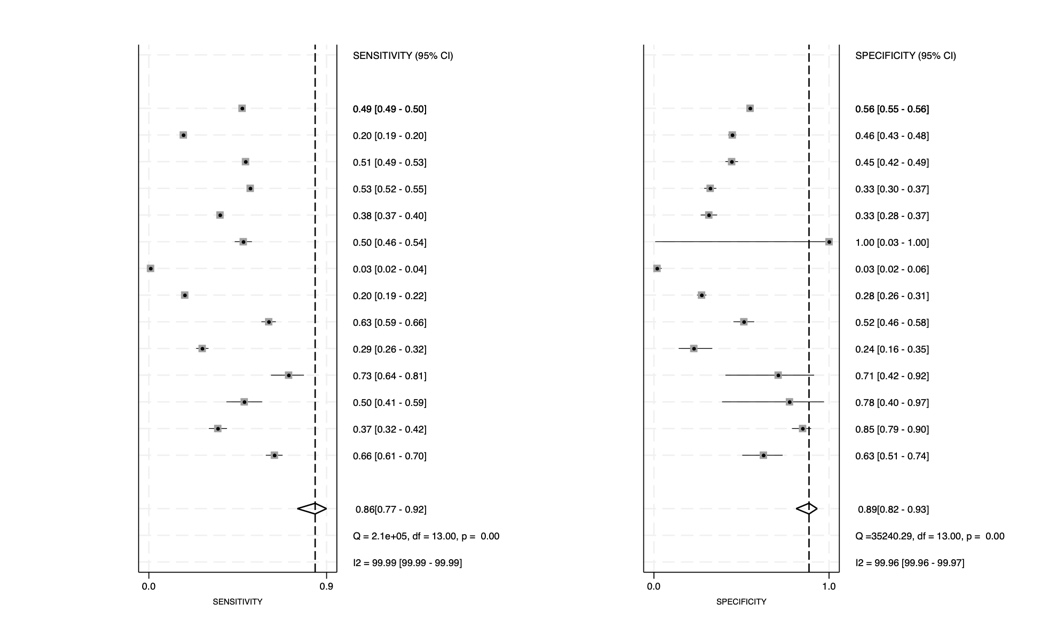


**A) forest plot for sensitivity and specificity** **after excluding studies with less than 500 sample size**


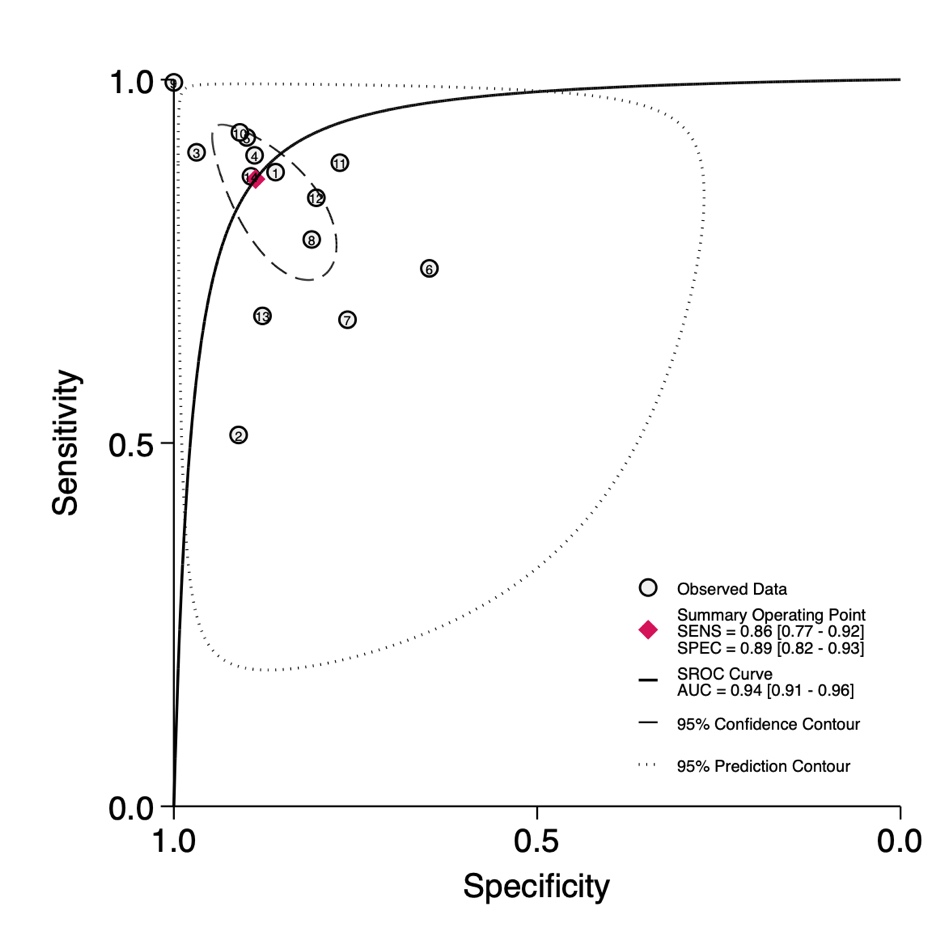


**B) HSROC curve after excluding studies with less than 500 sample size**
